# Complicated sea urchin-induced wound infection caused by *Vibrio* *alginolyticus* and *Staphylococcus lugdunensis* in a 14-year-old boy

**DOI:** 10.1099/jmmcr.0.005074

**Published:** 2016-12-19

**Authors:** Christoph André Bultmann, Jens-Oliver Steiß, Cornelia Langner, Birgit Benkert, Magdalena Havener, Uta Küsters, Stephan Georg Hühn-Lindenbein, Dietrich Mack

**Affiliations:** ^1^​ Bioscientia Institut für Medizinische Diagnostik GmbH, Mikrobiologie/Infektiologie, Ingelheim, Germany; ^2^​ University Children's Hospital, Giessen, Germany; ^3^​ Practice of Pediatrics, Fulda, Germany; ^4^​ Institute of Food Hygiene, Free University of Berlin, Berlin, Germany

**Keywords:** *Vibrio*, *Vibrio alginolyticus*, coagulase-negative staphylococci, *Staphylococcus lugdunensis*, wound, sea urchin

## Abstract

**Introduction:**

Wound infections with *Vibrio alginolyticus*, a Gram-negative bacterium found in all temperate oceans, are rarely reported. However, a rising incidence of wound infections caused by *V. alginolyticus* requires better knowledge about this infectious agent.

**Case presentation:**

We report the case of a 14-year-old boy suffering from a wound infection caused by *V. alginolyticus* and *Staphylococcus lugdunensis* after stepping on a sea urchin. Despite wound debridement and antibiotic therapy with cefaclor, the lesion did not heal over several weeks. After identification of the pathogens and antibiotic-susceptibility testing, antibiotic therapy was switched to ciprofloxacin, followed by trimethoprim/sulfamethoxazole. Two months after the accident the wound was re-epithelialized. Follow up after 6 months revealed a painful scar.

**Conclusion:**

Non-cholera vibrios like *V. alginolyticus* should be considered as possible causative agents in seawater-contaminated wounds. *S. lugdunensis* is a relevant pathogen in mixed wound infections. Early microbiological diagnosis and antibiotic-susceptibility testing is crucial to prevent therapeutic failure.

## Introduction

*Vibrio alginolyticus* is a Gram-negative halophilic bacterium found in temperate oceans all over the world. Most isolates show resistance to penicillins and second-generation cephalosporins (French *et al.*, 1989; Li *et al.*, 1999), and are capable of causing serious wound infections, even in association with only minor lesions (Gomez *et al.*, 2003; Reilly *et al.*, 2011).

Infections in humans are rarely reported, e.g. the incidence was only 0.048 per 100 000 population in the USA in 2011 (Conrad, 2013). Nevertheless, like other *Vibrio*-mediated infections there has been an increase in incidence over many years, possibly induced by an elevation of marine temperature due to global warming (Conrad, 2013; Vezzulli *et al.*, 2012).

Herein, we report the clinical course and follow up of a 14-year-old immunocompetent boy who was suffering from a mixed wound infection caused by *V. alginolyticus* and *Staphylococcus lugdunensis*. In the case of wounds acquired from contact with seawater or marine organisms, clinicians should be aware of *Vibrio* infections. Nevertheless, these infections are unknown to many physicians even in high-incidence countries (Osaka *et al.*, 2004) and require careful microbiological work-up.

## Case report

**Fig. 1.**
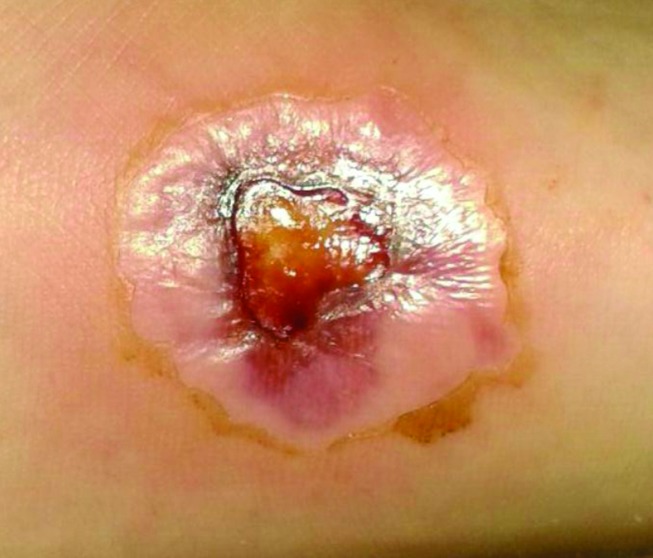
Lesion on the medial margin of the sole of the left foot 28 days after the accident. Diameter approximately 2 cm.

The patient was swimming in the Red Sea while on vacation in Egypt, near Hurghada, when he stepped on a sea urchin. He was injured on the medial plantar margin of his left foot (Fig. 1). The wound was treated immediately by the beach warden using hot oil and lemon juice. This treatment led to a minor burn with blistering.

Immediately afterwards, the patient was taken to the local hospital. There a burn blister on top of the wound was opened and disinfected with an iodine-containing ointment. The wound was covered with sterile dressing, which was changed daily. During the next 2 weeks, until the boy's departure from Egypt, the lesion became livid and kept oozing.

Back in Germany, the patient presented with the non-healing wound and an additional tonsillitis at a paediatric outpatient clinic. The wound was cleaned and empiric antibiotic therapy was started with cefaclor (500 mg three times daily). Seven days later, the patient returned to the clinic with continuing impaired wound healing, while the tonsillitis had resolved. A wound swab was submitted for microbiological analysis. The patient was then referred to the local hospital’s surgical outpatient clinic, where an extensive wound debridement was performed. Four days afterwards, the patient returned for a control examination and antibiotic therapy was changed to ciprofloxacin (200 mg twice daily).

Two bacterial isolates were obtained from the wound swab and identified as *V. alginolyticus* and *S. lugdunensis*. Numerous colonies were present up to the second (*V. alginolyticus*) and third (*S. lugdunensis*) streak area of the initial streak plates. After receiving antibiotic-susceptibility test results, therapy was switched to trimethoprim/sulfamethoxazole (160/800 mg twice daily) to which both isolates were susceptible. Antibiotic therapy with trimethoprim/sulfamethoxazole was continued for 20 days.

## Investigations

Aerobic and anaerobic cultures were performed using Columbia blood agar, chocolate agar, Columbia CNA agar, MacConkey agar and Schaedler agar with/without kanamycin, using standard microbiological procedures. A Gram-negative rod and coagulase-negative staphylococci were grown and identified to the species level using appropriate VITEK 2 ID cards (VITEK 2 GN and GP-cartridge; bioMérieux) as *V. alginolyticus* and *S. lugdunensis*. Antibiotic-susceptibility testing was performed using appropriate VITEK 2 cards, AST N263 and AST P619, respectively, and interpreted according to current Clinical and Laboratory Standards Institute (CLSI) guidelines (Table 1).

**Table 1.** MIC and antimicrobial-susceptibility test. Interpretive categories were according to the CLSI guidelines for *Vibrio* spp. (CLSI, 2015a) and *Staphylococcus* spp. with special regard to *S. lugdunensis* (CLSI, 2015b)

Antibiotic	*V. alginolyticus*		*S. lugdunensis*	
	MIC (mg l^−^^1^)	Interpretation	MIC (mg l^−^^1^)	Interpretation
Benzylpenicillin	nt*	nt	≥0.5	R
Ampicillin	≥32	R	nt*	nt
Ampicillin/sulbactam	≤2	S	nt*	nt
Oxacillin	nt*	nt	1	S
Cefuroxime	16	I	nt*	nt
Cefotaxime	≤1	S	nt*	nt
Ceftazidime	≤1	S	nt*	nt
Ciprofloxacin	≤0.25	S	nt	nt
Levofloxacin	≤0.12	S	≤0.12	S
Imipenem	≤0.25	S	–	S
Meropenem	≤0.25	S	–	S
Trimethoprim/sulfamethoxazole	≤20	S	≤10	S

I, Intermediate susceptibility; nt, not tested; R, resistant; S, susceptible.

*Testing not recommended for this species.

For confirmation of the *V. alginolyticus* identification, the strain was sub-cultured on *Vibrio* selective agar (thiosulfate-citrate-bile-sucrose agar; Becton-Dickinson) at 37 °C. After 24 h, colonies grew on the plates turning the colour of the agar to yellow as expected for *V. alginolyticus*. Additionally, matrix-associated laser desorption ionization-time of flight MS identification using a MALDI Biotyper (Bruker Daltonics) using software version 3.1 was performed, revealing *V. alginolyticus* with a score of 1.94. The next most closely related species was *Vibrio mytili* with a score of 1.84.

As the MALDI Biotyper revealed only an identification at the probable genus level, further confirmation was sought using 16S rRNA gene sequencing applying the method of Harmsen *et al.* (2003). The resulting 0.5 kbp amplicons were sequenced with a 3500xL Genetic Analyzer (Thermo Fisher Scientific). Using the curated database of EZbioCloud (Kim *et al.*, 2012) and criteria for microbial identification using DNA target sequences (CLSI, 2008),similarities larger than 99 % were found for numerous species of the genus *Vibrio,* including *V. alginolyticus,* without sufficient discrimination for identification at the species level. Similarly, the 16S rRNA gene sequence was analysed using blastn 2.2.26 and the DNA Database of Japan (DDBJ) due to its large number of well-documented *Vibrio* spp. genome sequences (http://ddbj.nig.ac.jp/blast/; Altschul *et al.*, 1997). More than 200 strains of *Vibrio* spp. shared the best-reached similarity of 98 % to our isolate, including 10 different species (*Vibrio fischeri, Vibrio parahaemolyticus, Vibrio harveyi, V. alginolyticus, Vibrio campbellii, Vibrio communis, Vibrio orientalis, Vibrio rotiferianus, Vibrio owensii* and '*Vibrio* *antiquarius*').

In the next step, additional multiplex PCR for the conserved transcriptional regulator genes *VptoxR, VctoxR* and *VvtoxR* (Osorio & Klose, 2000), according to Bauer & Rørvik (2007), was performed. There was a negative result for all *toxR* genes, leading to an exclusion of the species *V. parahaemolyticus, Vibrio cholerae* and *Vibrio vulnificus* from the identification.

**Fig. 2.**
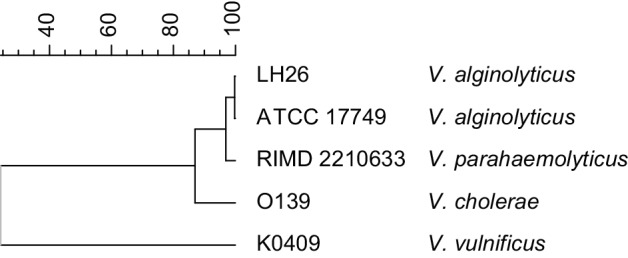
Phylogenetic tree including our isolate (*V. alginolyticus* LH26), *V. cholerae* O139 (NCBI reference sequence no. NZ_AWWA01000041.1), *V. parahaemolyticus* RIMD 2210633 (NCBI reference sequence no. NC_004603.1), *V. vulnificus* K0409 (GenBank accession no. EF064441.1) and *V. alginolyticus* ATCC 17749 (GenBank accession no. JN188438.1). The *x*-axis shows the percentage similarity.

Finally, *rpoB* sequencing applying the method and primers of Tarr *et al.* (2007) delivered two sequences of the *rpoB* gene (456 bp upstream, 528 bp downstream), which were analysed using DDBJ and blastn as described above (http://ddbj.nig.ac.jp/blast/; Altschul *et al.*, 1997). Our isolate showed identity of 100 % to more than 50 strains of *V. alginolyticus* (upstream) and 99 % to more than 200 strains of *V. alginolyticus* (downstream). There was one 99 % match with *V. harveyi* (upstream and downstream) and one with *V. parahaemolyticus* (upstream). The sequence data were analysed with Bionumerics (Applied Maths, version 7.1; Applied Maths) and compared to previously published sequences of the most common pathogenic *Vibrio* spp. in the National Center for Biotechnology Information (NCBI) database (http://www.ncbi.nlm.nih.gov/nuccore). To visualize the phylogenetic relationship, the unweighted pair group method with arithmetic mean based on multiple alignments between the *rpoB* sequences was used (Fig. 2).

Because of the former biochemical identification results and the large number of perfect homologies to strains identified in various taxonomic studies (Ki *et al.*, 2009; Oberbeckmann *et al.*, 2011), we accepted *V. alginolyticus* as the final identification. For coagulase-negative staphylococci, biochemical identification is widely used and commonly accepted (Becker *et al.*, 2014). Therefore, we accepted the Vitek 2-based identification of *S. lugdunensis* described above.

## Diagnosis

*V. alginolyticus* and *S. lugdunensis* co-infection of a sea urchin-induced wound.

## Outcome and follow-up

In the following months, wound healing continued slowly until the wound was epithelialized about 2 months later. When examined for follow-up 6 months after the initial accident, it was noticed that there remained an induration of the former wound with tenderness on palpation.

## Discussion

In this case, we identified three major reasons for the prolonged, complicated wound infection. First of all, insufficient first aid and the resulting burn necrosis led to an environment where *V. alginolyticus* and *S. lugdunensis* could survive repeated debridement and disinfection. Lack of protection because of burned skin enables secondary bacterial infections.

Second, *V. alginolyticus* is well known for its numerous chromosomal and plasmid-mediated antibiotic-resistance determinants (French *et al.*, 1989; Li *et al.*, 1999). Many of the expressed β-lactamases lead to resistance to ampicillin and second-generation cephalosporins, as seen in our isolate (Li *et al.*, 1999). Resistance to trimethoprim/sulfamethoxazole is commonly reported (Li *et al.*, 1999). Some isolates also show resistance to third-generation cephalosporins and fluoroquinolones, as reported by Ye *et al.* (2016). According to this evolution of antibiotic resistance and because of the typical mixed flora in chronic wound infections (like *S. lugdunensis* in our case; Altoparlak *et al.*, 2004), early antibiotic-susceptibility testing is important to prevent therapeutic failure.

Lastly, the presence of *S. lugdunensis* may have triggered the progression of the disease. Compared to many other coagulase-negative staphylococci, *S. lugdunensis* has higher pathogenic potential. It can cause serious infections, i.e. soft tissue and wound infections as well as infective endocarditis, and has to be considered as a relevant pathogen (Becker *et al.*, 2014).

When treating the patient, the chosen therapy in the hospital with ciprofloxacin was an appropriate choice for the infection. However, fluoroquinolone use in children is still off label for many indications (except, for example, cystic fibrosis), especially if there is an alternative treatment (Bradley *et al.*, 2011). Therefore, therapy was changed successfully to trimethoprim/sulfamethoxazole.

Altogether, this case and its course are an example of the need to consider *Vibrio-*mediated infections in similar circumstances. Even if it is a rare disease at present, a rising incidence has been observed, as indicated above. Warming of the oceans will probably make this a global trend as the first cases from northern shores suggest (Reilly *et al.*, 2011; Schets *et al.*, 2006). Identification of *V. alginolyticus* is less than straightforward and requires a combination of classical biochemical identification methods, as well as appropriate selective media and advanced molecular identification methodology.
